# Comparative microRNAs profile of *Schistosoma japonicum* male worms derived from single-sex and bisexual infections: Implications of the multifunctional role of microRNA

**DOI:** 10.1007/s00436-025-08489-x

**Published:** 2025-04-24

**Authors:** Haoran Zhong, Danlin Zhu, Bowen Dong, Luobin Wu, Ke Lu, Zhiqiang Fu, Jinming Liu, Guiquan Guan, Yamei Jin

**Affiliations:** 1 National Reference Laboratory for Animal Schistosomiasis, Key Laboratory of Animal Parasitology of Ministry of Agriculture and Rural Affairs, Shanghai Veterinary Research Institute, Chinese Academy of Agricultural Sciences, Shanghai, P.R. China; 2 College of Life Sciences, Shanghai Normal University, Shanghai, P.R. China; 3 State Key Laboratory for Animal Disease Control and Prevention, College of Veterinary Medicine, Lanzhou University, Lanzhou Veterinary Research Institute, Chinese Academy of Agricultural Sciences, Lanzhou, Gansu China; 4 Key Laboratory of Veterinary Parasitology of Gansu Province, Gansu Province Research Center for Basic Disciplines of Pathogen Biology, Lanzhou, Gansu China

**Keywords:** *Schistosoma japonicum*, MiRNA, Single-sex male worms, Mated male worms

## Abstract

*Schistosoma japonicum* is a dioecious parasite that requires constant pairing between male and female worms for female maturation and egg production. MicroRNAs (miRNAs) play crucial roles in regulating various biological processes, including parasite development and host–pathogen interactions, but their functions in schistosomes remain largely unexplored. This study aimed to investigate the differentially expressed miRNAs (DEMs) between mated male (MM) and single-sex male (SM) worms to gain insights into their regulatory roles in schistosome reproduction. Total RNA was extracted from 28-day-old MM and SM worms, followed by small RNA sequencing to identify DEMs. Bioinformatics analyses were used to predict the biological functions of DEM target genes. Comparative analysis with previously published miRNA datasets helped identify potentially significant miRNAs. Quantitative PCR (qPCR) validated the expression of selected miRNAs and mRNA levels of some target genes. A total of 20 DEMs were identified, with 9 upregulated in MM worms and 11 in SM worms. These DEMs may regulate processes such as intracellular transport, RNA processing, and cellular homeostasis. The study provides novel insights into the biological differences between SM and MM worms, suggesting that these miRNAs could be key regulators of parasite development and host adaptation, with potential diagnostic and therapeutic applications in schistosomiasis.

**Graphical abstract:**

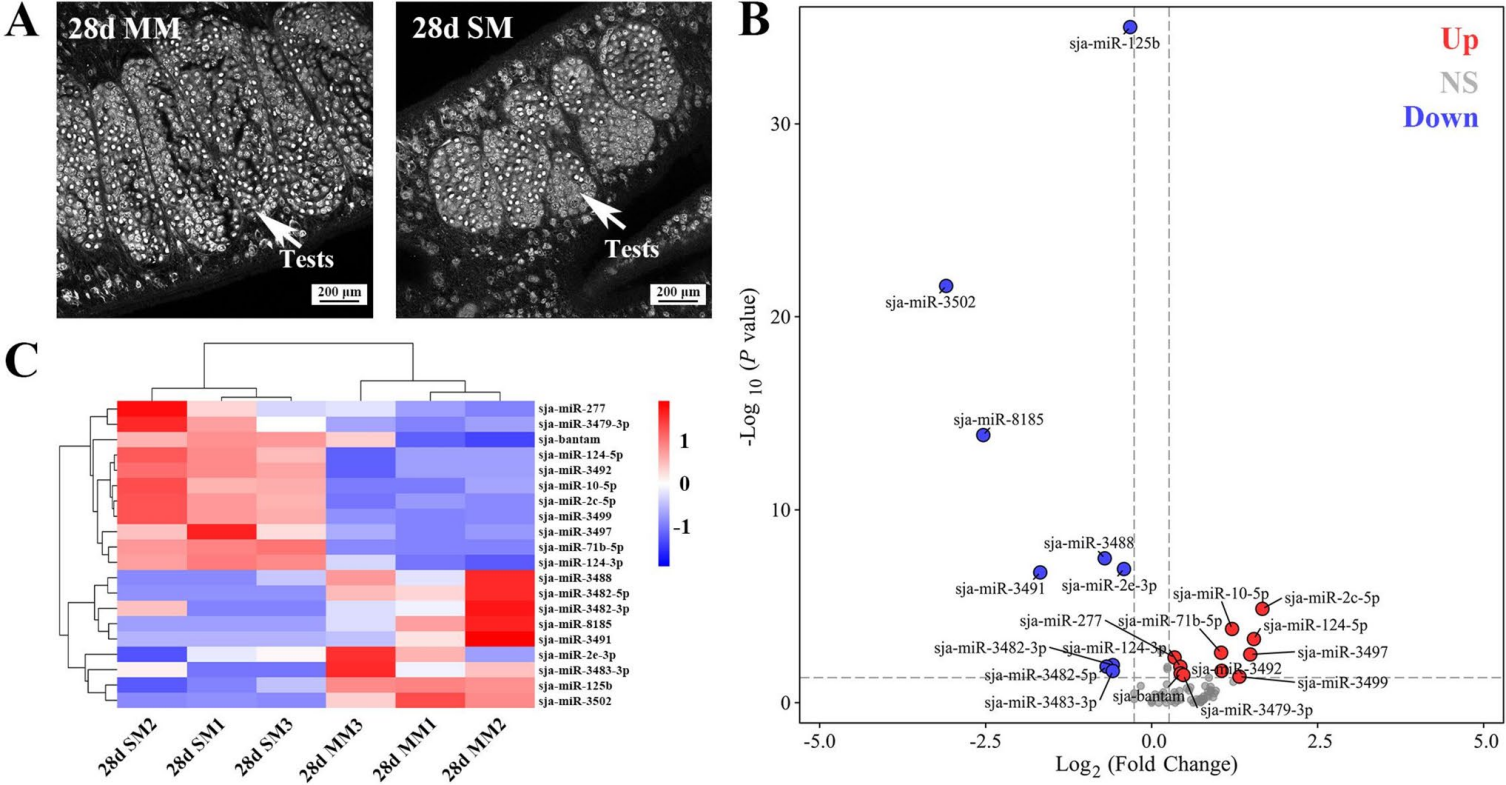

**Supplementary Information:**

The online version contains supplementary material available at 10.1007/s00436-025-08489-x.

## Introduction

Schistosomes are unique among parasitic flatworms due to their dioecious nature, requiring constant pairing between male and female worms to ensure female maturation and egg production (McManus et al. 2018). Unlike other trematodes, where reproduction can occur hermaphroditically, schistosome females depend on male worms for structural support and the provision of essential nutrients and signals. Male worms clasp females within the gynecophoral canal, facilitating prolonged pairing, which is crucial for the activation of female reproductive organs and continuous egg laying. Various signaling pathways have been implicated in this complex reproductive process, highlighting its regulatory intricacy and the involvement of multiple molecular factors (Chen et al. 2022). However, despite extensive studies, the precise mechanisms governing schistosome reproductive development remain largely elusive.

MicroRNAs (miRNAs) are small, non-coding RNA molecules that post-transcriptionally regulate gene expression and play essential roles in various biological processes, including development, metabolism, and host–pathogen interactions (Xue et al. 2008). In schistosomes, miRNAs have been implicated in the regulation of reproductive development and host-parasite interactions (Xue et al. 2008; Simões et al. 2011; Stroehlein et al. 2018). Recent studies have identified key schistosome miRNAs involved in parasite adaptation, growth, and sexual maturation. For instance, *Schistosoma japonicum* sja-bantam (Zhu et al. 2016), sja-miR- 1 (Sun et al. 2023), sja-miR- 124 - 3p (Zhou et al. 2022), sja-miR- 31 (Zheng et al. 2024) have been demonstrated to regulate female reproductive development and ovary maturation by targeting genes associated with egg production and reproductive tissue maintenance. The suppression of these miRNAs in female worms led to significant morphological alterations in the ovary, emphasizing their crucial roles in sexual development. Moreover, schistosome-derived extracellular vesicles (EVs) enriched with specific miRNAs such as sja-miR- 71a (Wang et al. 2020), sja-miR- 2162 (He et al. 2020), and sja-let- 7 (Zhong et al. 2024) have been shown to play pivotal roles in host-parasite interactions. These EV-associated miRNAs modulate host immune responses and contribute to the development of fibrosis in infected organs. Despite these significant advancements, the functional roles of many schistosome miRNAs remain poorly understood, necessitating further investigation to fully elucidate their biological significance.

*S. japonicum* mated male (MM) and single-sex male (SM) worms exhibit distinct physiological differences that provide valuable insights into the molecular mechanisms underlying schistosome reproductive biology (Zhong et al. 2022). Previous proteomic studies by our group have demonstrated that MM worms, which develop in the presence of female worms, exhibit upregulation of proteins related to reproductive function, metabolic activity, and host adaptation. These worms are physiologically primed for reproductive processes and show significant changes in signaling pathways involved in gametogenesis, nutrient acquisition, and energy metabolism. In contrast, SM worms, which develop without female pairing, prioritize cellular homeostasis and survival strategies, exhibiting a metabolic profile focused on growth and maintenance rather than reproduction. These differences highlight the critical influence of female worms on male reproductive physiology (Zhong et al. 2022). Despite well-established protein-level differences, the regulatory roles of miRNAs in these processes remain largely unexplored. Given their well-documented functions in post-transcriptional regulation, miRNAs are likely to play significant roles in orchestrating the biological differences observed between MM and SM worms.

This study analyzed differentially expressed miRNAs (DEMs) between MM and SM worms to identify key miRNAs that may regulate upstream biological processes, potentially influencing downstream effects. Given the role of miRNAs in regulating critical pathways like reproductive development and host-parasite interactions, the differences between MM and SM worms offer insights into schistosome biology. The findings contribute to the understanding of miRNA functionality in schistosomes, providing new perspectives on parasite adaptation and reproductive biology, and may lay the groundwork for novel diagnostic and therapeutic strategies for schistosomiasis.

## Materials and methods

### Animals and parasites

Specific-pathogen-free (SPF) male BALB/c mice, aged 6–7 weeks and weighing 18 ± 2 g, were obtained from Shanghai Jiesijie Laboratory Animal Co., Ltd. (Shanghai, China) and maintained in SPF-standard animal facilities at the Shanghai Veterinary Research Institute. The Chinese strain of *S. japonicum* used in this study was supplied by the National Reference Laboratory for Animal Schistosomiasis. Cercariae were obtained by exposing infected *Oncomelania hupensis* snails to light, and their gender was determined via PCR as described in patent CN101597645. This method employs a set of specificity detection primers designed based on a female-specific sequence of *S. japonicum*. The upstream primer (Tsexu2: 5'-ACGTTAGATACTGCTGTTCA- 3') and downstream primer (Tsexd2: 5'-ATATTGTTCCAAGTACGCAT- 3') amplify female-specific DNA, allowing for sex identification. PCR products are analyzed via agarose gel electrophoresis under ultraviolet light, where a distinct amplification band appears only in female samples, ensuring a rapid, specific, and sensitive diagnostic approach for sex determination in *S. japonicum*. Then, BALB/c mice were infected percutaneously with either single-sex or mixed-sex cercariae. At 28 days post-infection (dpi), the mice were euthanized, and worms were collected via hepatic-portal perfusion.

### Confocal microscopy of 28-day MM and SM worms

The 28-day SM worms were collected via hepatic-portal perfusion, while the 28-day MM worms were manually separated. Both groups were fixed for 15 h, stained with carmine (Sigma-Aldrich, USA) at 37 °C for 12 h, and cleared in 70% acidic ethanol. Dehydration was performed through a graded ethanol series (80%, 95%, and 100%) for 40 min each. The worms were then preserved in neutral balsam (Solarbio, China), mounted on glass slides, and visualized using a confocal scanning microscope (Nikon, Japan).

### Total RNA isolation, small RNA library construction, and sequencing

Total RNA was extracted using TRIzol reagent (Invitrogen, USA) following the manufacturer’s instructions. RNA concentration was measured with a Qubit 4.0 fluorometer (Invitrogen, USA), and quality was assessed via denaturing agarose gel electrophoresis. Upon confirming RNA integrity, miRNA libraries were constructed using the VAHTS® Small RNA Library Prep Kit for Illumina (Vazyme, China) and sequenced on the Illumina NovaSeq 6000 platform. RNA enrichment, library preparation, sequencing, and data analysis were performed by Shanghai Xu Ran Biotechnology Co., Ltd.

Raw reads were filtered to remove poly-N sequences, low-quality reads, and those shorter than 15 nucleotides or longer than 32 nucleotides. Clean reads were aligned to the *S. japonicum* V3 genome assembly (SRA accession: PRJNA739049) using Bowtie (v1.0.0) (Langmead and Salzberg 2012), with the following settings: -q for FASTQ format input, -S for SAM format output, -v 1 to allow one mismatch, and -p 10 to utilize 10 threads for alignment. miRNA expression levels for each sample were quantified using the counts per million (CPM) algorithm. Differential expression analysis was conducted with the DESeq2 R package (v1.22.1) (Wang et al. 2010), using the default parameters. miRNAs with a fold change (FC) of ≥ 1.2 and a P-value of ≤ 0.05 were considered as DEMs.

### Predictions and bioinformatics analyses of target genes of DEMs

The target genes of DEMs were predicted using the miRanda tool (v3.3a) (Peterson et al. 2014),with the following settings: -sc 140 for the scoring threshold, -en − 30 for the energy threshold, -quiet to suppress output, and -strict for stricter filtering rules. Subsequently, TopGO (https://www.bioconductor.org/packages/release/bioc/html/topGO.html) (Conesa et al. 2005) and Kyoto Encyclopedia of Genes and Genomes (KEGG) database (https://www.genome.jp/kegg/pathway.html) (Kanehisa and Goto 2000) were performed to explore the enrichment analyses of these target genes. Pathways showing significant enrichment were identified based on a threshold of *P* < 0.05.

### Quantitative reverse transcription PCR (qRT-PCR)

Total RNA from MM and SM was extracted as mentioned above. To remove any potential DNA contamination, total RNA was treated with DNase I (RNase-free) (Takara, Japan) during the extraction process. For miRNA analysis, first-strand cDNA synthesis was carried out using the miRNA First Strand cDNA Synthesis Kit (Stem-loop Method) (Sangon, China) with a stem-loop RT primer specific to each miRNA. The synthesized cDNA was subsequently used for qPCR with the miRNA qPCR Kit (SYBR Green Method) (Sangon, China), and the relative expression levels of miRNAs were normalized to U6, serving as the endogenous control (Yu et al. 2019a). qPCR was conducted on the LightCycler 96 system with an initial denaturation at 95℃ for 60 s, followed by 40 cycles at 95℃ for 5 s and 62℃ for 30 s, with a melting curve analysis at 95℃ for 10 s, 65℃ for 60 s, and 97℃ for 1 s. For mRNA analysis, reverse transcription was performed with the Hifair III 1 st Strand cDNA Synthesis SuperMix for qPCR kit (Yeasen, China), and cDNA was used for qPCR amplification with Hieff qPCR SYBR Green Master Mix (Yeasen, China). mRNA levels were normalized to the internal reference gene 26S proteasome non-ATPase regulatory subunit 4 (PSMD4) (Liu et al. 2012). The qPCR conditions were as follows: pre-incubation at 95℃ for 60 s, followed by 40 cycles at 95℃ for 5 s and 60℃ for 30 s, with a final melting curve analysis. Relative expression levels of miRNAs and mRNAs were calculated using the 2^−ΔΔCt^ method (Livak and Schmittgen 2001). Primer sequences are listed in Table S1.

### Comparative analysis of DEMs across databases

An intersection analysis was performed to compare the DEMs identified in the present research with those previously reported for *S. japonicum* under various conditions. The comparison included DEMs from female worms derived from single-sex infections (SF) and bisexual infections (MF) (Han et al. 2020), as well as worms obtained from different host sources, including SCID mice (Liu et al. 2022), BALB/c mice (Han et al. 2015a, b), Wistar rats (Han et al. 2015b), *Microtus fortis* (Han et al. 2015a), water buffalo (Yu et al. 2019b), and yellow cattle (Yu et al. 2019b).

For further analysis, worms obtained from single-sex male and female infections (SM and SF), SCID mice, Wistar rats, *Microtus fortis*, and water buffalo were defined as “immature worms”, whereas worms derived from bisexual infections (MM and MF), BALB/c mice, and yellow cattle were categorized as “mature worms”. Common miRNAs were identified by intersecting the DEMs from immature and mature worm groups. To further validate their expression patterns across different developmental stages of *S. japonicum*, the identified miRNAs were compared against an additional small RNA sequencing database (Yu et al. 2019a). The temporal expression dynamics of these miRNAs in male and female *S. japonicum* worms were analyzed at various developmental stages, ranging from 14 to 28 dpi, and the results were visualized using line charts.

### Statistical analysis

Data analysis was performed using SPSS 25.0 software (SPSS Inc., USA), and the results are expressed as the mean ± standard deviation (SD) based on three independent biological replicates. Statistical significance was assessed using Student’s *t*-test, with a significance threshold set at *P* < 0.05.

## Results and discussion

### Morphological differences and DEM analysis of 28-day MM and SM

**Fig. 1:**
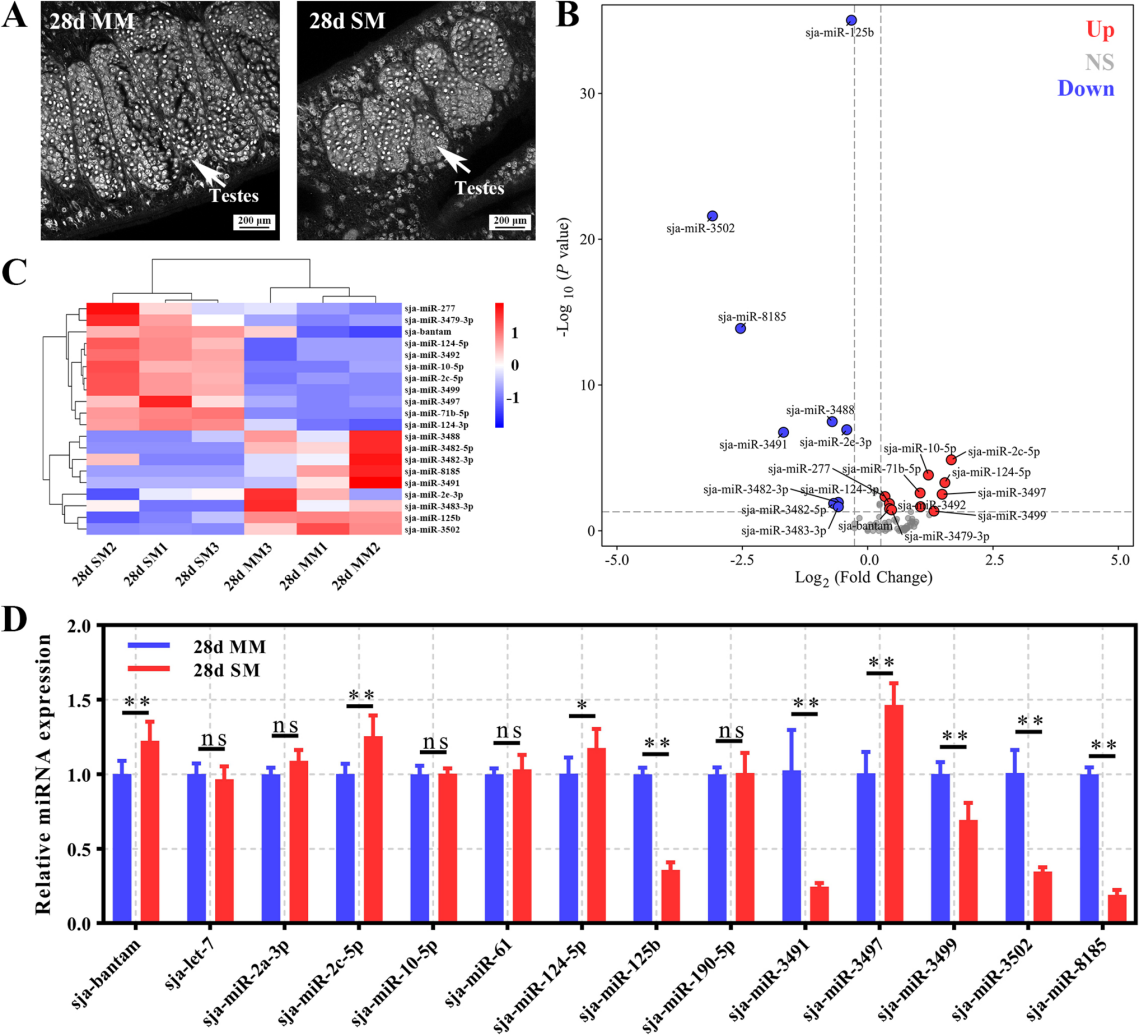
Morphological and differential expression analysis of miRNAs between MM and SM worms. **A** Representative images of testicular structures in 28-day MM and SM worms, Scale bar: 200 μm. **B** Volcano plot illustrating DEMs between MM and SM worms (**C**) Heatmap showing hierarchical clustering of DEMs in MM and SM worms. **D** Validation of selected miRNAs by qPCR. All experiments were performed in triplicate and are expressed as the mean ± SD. Significant differences are indicated (**P < 0.01, *P < 0.05, ns *P* > 0.05)

The morphological differences between MM and SM are not easily distinguishable through visual inspection (Shi et al. 2014). Confocal microscopy revealed slight differences in testis size, with MM worms exhibiting slightly larger testes than SM worms (Zhong et al. 2022) (Fig. 1A). An FC threshold of 1.2 was applied to capture subtle but biologically relevant differences in miRNA expression between MM and SM worms.

The sequencing data exhibited strong reliability and consistency across replicates. Read length distribution analysis confirmed that most small RNA reads fell within the expected size range (Fig. S1A). Boxplot analysis of log-transformed CPM indicated uniform distribution across all samples, ensuring consistent sequencing depth and quality (Fig. S1B). Principal component analysis (PCA) revealed distinct clustering of MM and SM groups, highlighting clear differences in miRNA expression profiles (Fig. S1C). A total of 9 miRNAs were upregulated in MM worms, while 11 showed higher expression in SM worms (Fig. 1B and 1 C, Table S2). To validate the sequencing results, 14 miRNAs were randomly selected for qPCR analysis, confirming the expression patterns of 12 miRNAs, while sja-miR- 10 - 5p and sja-miR- 3499 showed discrepancies with sequencing data (Fig. 1D).

### Functional analysis and validation of target genes

**Fig. 2:**
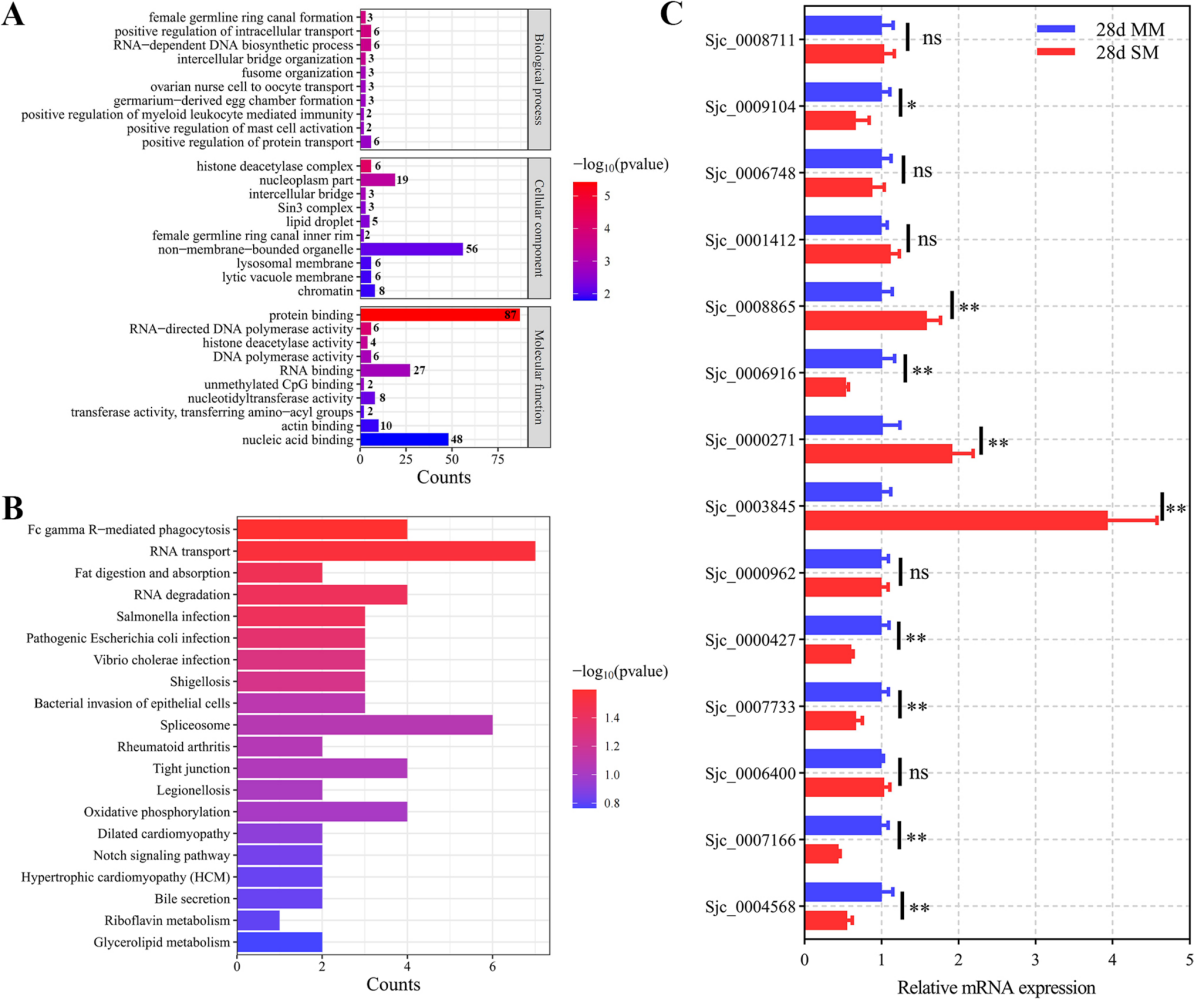
Functional enrichment analysis and qPCR validation of target genes. **A** GO enrichment analysis of DEM target genes categorized into biological processes, cellular components, and molecular functions. **B** KEGG pathway enrichment analysis of DEM target genes. **C** qPCR validation of selected target genes in MM and SM worms. All experiments were performed in triplicate and are expressed as the mean ± SD. Significant differences are indicated (**P < 0.01, *P < 0.05, ns *P* > 0.05)

To investigate the biological functions of the DEMs, target gene prediction was conducted followed by GO and KEGG enrichment analyses. All target genes were presented in Table S3. The GO analysis suggested that the target genes may be involved in processes like female germline ring canal formation and intracellular transport, contributing to reproductive development and cellular organization. Enrichment in RNA-dependent DNA biosynthesis suggests a role in genetic material maintenance and cellular replication (Fig. 2A, Table S4). Genes enriched in intercellular bridges and non-membrane-bounded organelles may be involved in cellular communication and structural organization, essential for parasite growth and adaptation. The molecular function analysis indicated that these miRNAs might regulate protein and RNA binding activities, influencing post-transcriptional regulation and parasite development (Fig. 2A, Table S4). KEGG pathway analysis revealed participation in RNA transport, RNA degradation, and metabolic processes like oxidative phosphorylation and riboflavin metabolism (Fig. 2B, Table S5). These findings suggest that DEMs may regulate RNA stability and metabolic functions critical for parasite survival and energy production. Additionally, the identified target genes may highlight physiological differences between SM and MM worms, offering insights into their distinct adaptive and developmental strategies.

Our previous proteomic analysis of SM and MM worms revealed that, following pairing with female worms, MM worms exhibited upregulation of proteins linked to reproductive functions, while SM worms prioritized maintaining fundamental physiological and metabolic processes (Zhong et al. 2022). In this study, a subset of target genes from DEMs was selected for qPCR validation to investigate their expression patterns (Fig. 2C, Table S6). Specifically, Sjc_0004568 (Ras-related protein Rab- 4 A), involved in intracellular and vesicle transport (Qadeer et al. 2021), was upregulated in MM worms, indicating a higher demand for protein trafficking and cellular communication, potentially enhancing male-to-female signaling for reproductive regulation (Chen et al. 2022).

In contrast, the significant upregulation of Sjc_0000271 (Elongation Factor 1-Alpha) and Sjc_0003845 (DNA replication licensing factor mcm7-A) in SM worms suggests a focus on preserving cellular homeostasis and supporting growth processes rather than reproduction (Schüssler et al. 1997; Song et al. 2022). The elevated expression of Elongation Factor 1-Alpha, a key regulator of protein synthesis and cell growth, may reflect that SM worms prioritize self-sustenance over reproductive adaptation. Additionally, the higher expression of Sjc_0006916 (T-complex protein 1 subunit delta) in SM worms indicates an adaptive response to reinforce cytoskeletal organization and metabolic stability, critical for survival in a single-sex environment (Lu et al. 2020) (Fig. 2C). Conversely, lower expression in MM worms may suggest a physiological shift towards reproductive processes due to the presence of female worms.

In summary, the analysis of DEM target genes suggests their involvement in key biological processes, cellular structures, and metabolic pathways that may contribute to the distinct physiological states of SM and MM worms. However, further experimental validation is needed to fully understand the roles of these miRNAs and their target genes in the development and adaptation of S. japonicum male worms.

### Comparative analysis of DEMs across databases

**Fig. 3:**
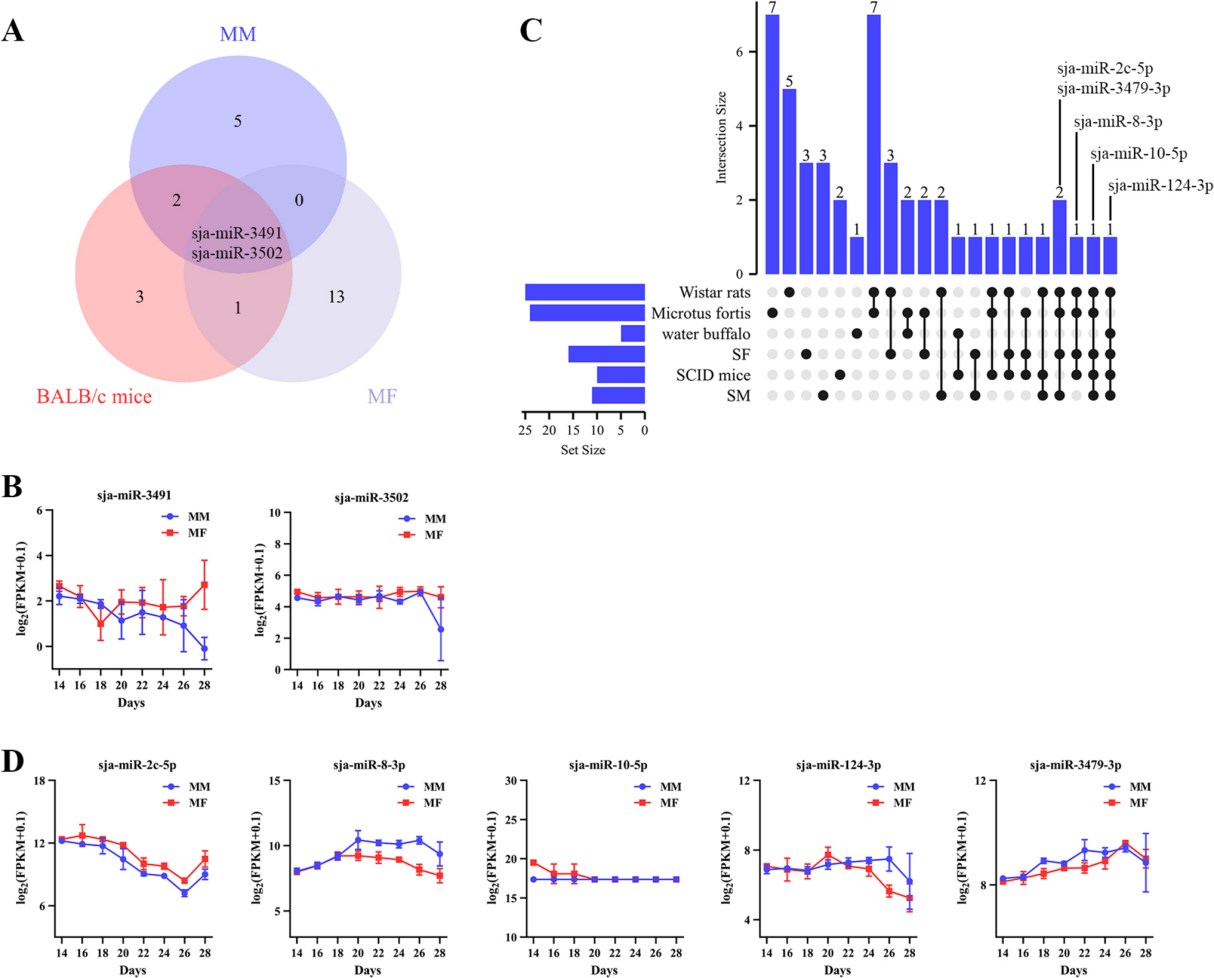
Comparative analysis of identified miRNAs across different schistosome datasets. **A** Venn diagram showing the overlap of DEMs among MM, MF, and BALB/c mice-derived worms. **B** Expression dynamics of sja-miR- 3491 and sja-miR- 3502 over the 14–28 dpi period in MM and MF worms. **C** UpSet plot illustrating the intersection of DEMs among SM, SF, SCID mice, Wistar rats, *Microtus fortis*, and water buffalo. **D** Expression dynamics of sja-miR- 2c- 5p, sja-miR- 8- 3p, sja-miR- 10 - 5p, sja-miR- 124 - 3p, and sja-miR- 3479 - 3p over the 14–28 dpi period in MM and MF worms

In this study, worms from single-sex male and female infections (SM and SF), SCID mice, Wistar rats, *Microtus fortis*, and water buffalo were categorized as “immature worms,” while worms from bisexual infections (MM and MF), BALB/c mice, and yellow cattle were classified as “mature worms.” Comparative analysis identified sja-miR- 3491 and sja-miR- 3502 in the mature worm group (Fig. 3A, Table S7). Previous small RNA sequencing showed that these miRNAs exhibit low but stable expression between 14–28 dpi in both male and female worms (Fig. 3B) (Yu et al. 2019a). This stability suggests they may play a role in maintaining parasite homeostasis. However, limited research on these miRNAs warrants further investigation of their biological functions.

The immature worm group yielded sja-miR- 10 - 5p, sja-miR- 2c- 5p, sja-miR- 8- 3p, sja-miR- 124 - 3p, and sja-miR- 3479 - 3p (Fig. 3C, Table S8), which are associated with various biological functions of *S. japonicum*. Expression analysis revealed distinct patterns across developmental time points, suggesting their involvement in multiple biological processes (Fig. 3D). Notably, sja-miR- 124 - 3p has been well studied for its role in growth, development, and reproduction (Zhou et al. 2022). It is expressed at higher levels in schistosomes from non-permissive hosts (*Microtus fortis*, rats, and water buffalo) compared to suitable hosts like mice and yellow cattle, suggesting its role in parasite adaptation to less favorable environments. Additionally, its higher expression in SF worms compared to MF worms indicates a role in sexual development and reproductive regulation. Overexpression of sja-miR- 124 - 3p in infected mice reduced hepatic egg burden and granuloma size, suggesting its link to reproductive capacity or embryonic viability. Target prediction identified DEAD-box ATP-dependent RNA helicase 1 (DDX1) as a direct target of sja-miR- 124 - 3p. Silencing of SjDDX1 led to a reduction in worm and egg burdens, indicating that sja-miR- 124 - 3p regulates critical biological processes essential for parasite survival and reproduction (Zhou et al. 2022).

Similarly, sja-miR- 8- 3p and sja-miR- 10 - 5p have been implicated in various developmental and regulatory processes, with growing evidence suggesting their potential roles in parasite development (Liu et al. 2022). The miR- 8- 3p family, well known for its regulatory functions in insects. For instance, the conserved miR- 8- 3p has been shown to coordinate the expression of V-ATPase subunits, thereby regulating ecdysone biosynthesis during metamorphosis in *Drosophila*, and playing a crucial role in the development and metamorphosis of the red flour beetle *Tribolium castaneum* (Wu et al. 2019; Lim et al. 2020). These findings suggest that miR- 8- 3p may have a conserved role in parasite developmental pathways. Moreover, both sja-miR- 8- 3p and sja-miR- 10 - 5p have been identified as regulators of key molecules within reproductive and developmental signaling pathways, further supporting their potential roles in parasite growth, adaptation, and reproductive processes (Zhu et al. 2016).

Notably, sja-miR- 2c- 5p and sja-miR- 3479 - 3p exhibit distinct sex-specific expression patterns, with sja-miR- 2c- 5p predominantly expressed in female worms and sja-miR- 3479 - 3p primarily detected in male worms (Zhu et al. 2016). This differential expression pattern suggests their potential involvement in the reproductive development and sexual differentiation of *S. japonicum.* Interestingly, both miRNAs have been identified in host serum and *S. japonicum* EVs, highlighting their potential as promising diagnostic biomarkers (Hoy et al. 2014; Liu et al. 2019). Studies have demonstrated that sja-miR- 2c- 5p maintains consistently high levels in the serum of infected hosts, although its correlation with infection intensity is relatively weaker compared to conventional diagnostic methods, such as the Kato-Katz test (Mu et al. 2019). Nevertheless, the stable presence of sja-miR- 2c- 5p in serum suggests its potential utility as a promising diagnostic tool.

In contrast, sja-miR- 3479 - 3p has been strongly correlated with hepatic egg burden and fibrosis severity in infected mice, highlighting its potential as a valuable biomarker for monitoring disease progression (Cai et al. 2015). Its abundant presence in host serum further suggests a critical role in parasite-host interactions, potentially contributing to immune modulation and facilitating parasite adaptation within the host environment. Recent studies have identified a homolog of this miRNA in the carcinogenic liver fluke *Opisthorchis viverrini*, known as ovi-miR- 3479a, which has been shown to target cancer-associated pathways and significantly promote cell proliferation in human cholangiocyte cell lines (Tan et al. 2024). These findings imply potential functional conservation across parasitic species, indicating the importance of sja-miR- 3479 - 3p in parasite biology and host–pathogen interactions, warranting further investigation.

Despite advances in schistosome research, the biological functions of only a limited number of *Schistosoma* miRNAs have been thoroughly characterized (Zhu et al. 2016; Zhou et al. 2022; Sun et al. 2023; Zheng et al. 2024), leaving substantial gaps in our understanding of their roles. In this study, attention was focused on DEMs between MM and SM worms, which exhibit minor morphological differences. However, the identified DEMs are suggested to play crucial upstream regulatory roles, potentially leading to significant downstream biological effects. The findings of this study provide valuable insights into the molecular mechanisms governing schistosome development and adaptation. The present study is primarily based on the relatively more-characterized *S. japonicum* miRNA database. In *S. mansoni, S. haematobium* and *S. mekongi,* the miRNAs profiles were characterized, research on the specific functions of individual miRNAs in these species is expected to be further developed in the future (Simões et al. 2011; Stroehlein et al. 2018; Sivapornnukul et al. 2024). Comprehensive investigations into these key miRNAs are essential to elucidate the regulatory networks underlying *Schistosoma* reproductive development, pathogenesis, and host interactions. A deeper exploration of these miRNAs may contribute to an improved understanding of schistosome biology and facilitate the development of novel diagnostic and therapeutic strategies for schistosomiasis control.

## Supplementary Information

Below is the link to the electronic supplementary material.

Supplementary file1 Fig. S1 Quality assessment of small RNA sequencing data. (A) Read length distribution of small RNA sequencing reads across different samples. (B) Boxplot of log2 counts per million (CPM) values for each sample. (C) Principal component analysis plot illustrating the clustering of samples based on their expression profiles (JPG 376 KB)

Supplementary file2 (DOCX 22 KB)

Supplementary file3 (DOCX 20 KB)

Supplementary file4 (XLSX 47 KB)

Supplementary file5 (XLSX 34 KB)

Supplementary file6 (XLSX 23 KB)

Supplementary file7 (XLSX 495 KB)

Supplementary file8 (XLSX 11 KB)

Supplementary file9 (XLSX 12 KB)

## Data Availability

The datasets presented in this study can be found in online repositories. The names of the repository/repositories and accession number can be found below: CNGB Sequence Archive of China National GeneBank DataBase, CNP0006748. Other data that support the findings of this study are available in the paper and its supplementary materials.
