# Supplementary figures and images for: Comparative microRNAs profile of Schistosoma japonicum male worms derived from single-sex and bisexual infections: Implications of the multifunctional role of microRNA
Source: Parasitol Res. 2025 Apr 24;124(4):43. doi: 10.1007/s00436-025-08489-x (PMC12021732; doi:10.1007/s00436-025-08489-x)

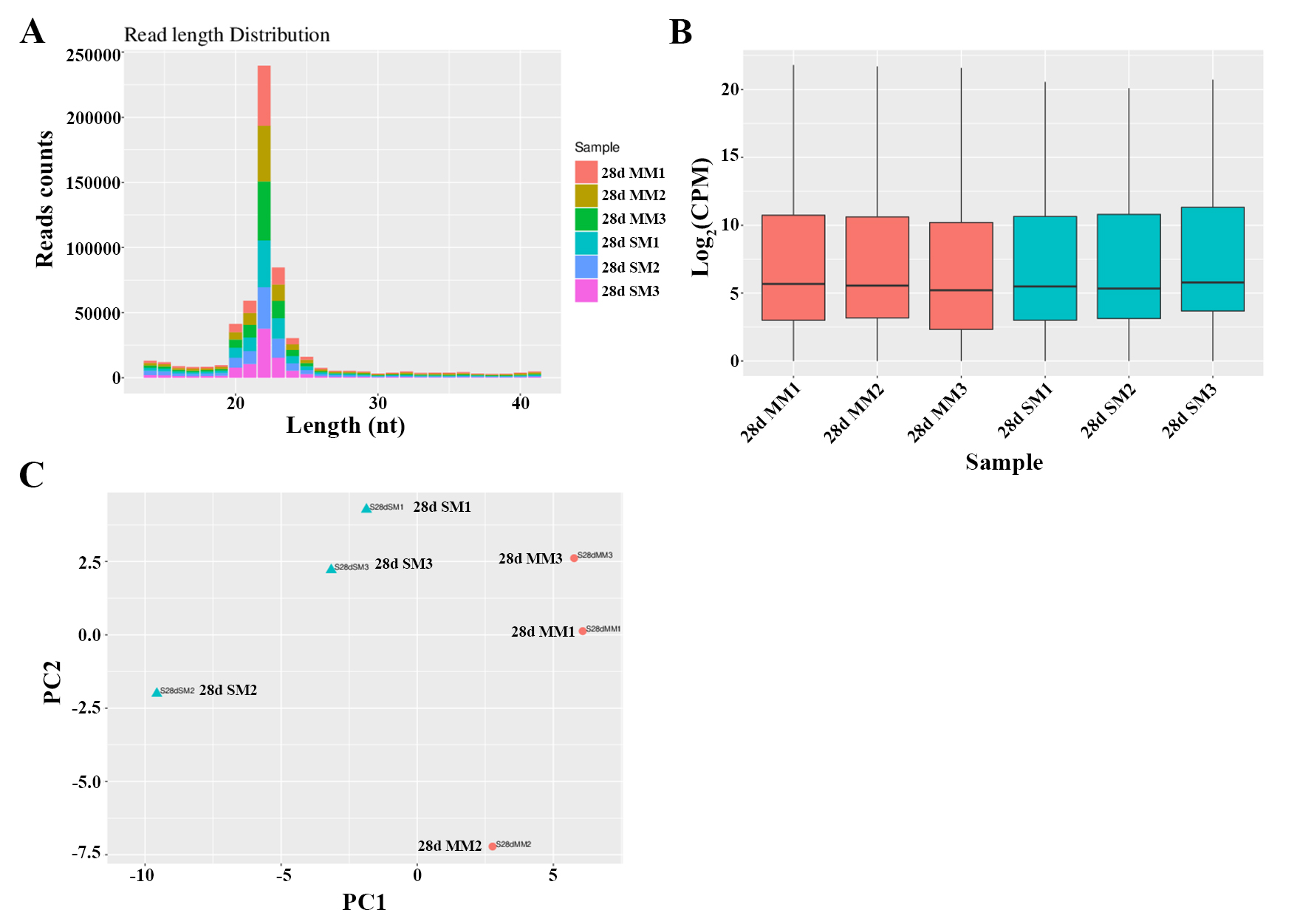

Supplement: Supplementary file 1 — Supplementary file1 Fig. S1 Quality assessment of small RNA sequencing data. (A) Read length distribution of small RNA sequencing reads across different samples. (B) Boxplot of log2 counts per million (CPM) values for each sample. (C) Principal component analysis plot illustrating the clustering of samples based on their expression profiles (JPG 376 KB) [file 436_2025_8489_MOESM1_ESM.jpg]
