# Supplementary material for: Comparative microRNAs profile of Schistosoma japonicum male worms derived from single-sex and bisexual infections: Implications of the multifunctional role of microRNA
Source: Parasitol Res. 2025 Apr 24;124(4):43. doi: 10.1007/s00436-025-08489-x (PMC12021732; doi:10.1007/s00436-025-08489-x)
Supplement: Supplementary file 2 — Supplementary file2 (DOCX 22 KB) [file 436_2025_8489_MOESM2_ESM.docx]

**Comparative microRNAs profile of *Schistosoma japonicum* male worms derived from single-sex and bisexual infections: Implications of the multifunction role of microRNA**

Haoran Zhong^1^, Danlin Zhu^1^, Bowen Dong^1^, Luobin Wu^1,2^ Ke Lu^1^, Zhiqiang Fu^1^, Jinming Liu^1^, Guiquan Guan^3,4^, Yamei Jin^1,*^

^1^ National Reference Laboratory for Animal Schistosomiasis, Key Laboratory of Animal Parasitology of Ministry of Agriculture and Rural Affairs, Shanghai Veterinary Research Institute, Chinese Academy of Agricultural Sciences, Shanghai, P.R. China

^2^ College of Life Sciences, Shanghai Normal University, Shanghai, P.R. China

^3^ State Key Laboratory for Animal Disease Control and Prevention, College of Veterinary Medicine, Lanzhou University, Lanzhou Veterinary Research Institute, Chinese Academy of Agricultural Sciences, Lanzhou, Gansu, China

^4^ Key Laboratory of Veterinary Parasitology of Gansu Province, Gansu Province Research Center for Basic Disciplines of Pathogen Biology, Lanzhou, Gansu, China

^*^ Correspondence: [yameijin@shvri.ac.cn](mailto:yameijin@shvri.ac.cn)

**Table S1. Primers used in the experiment.**

| Primer | Sequences (5’-3’) |
| --- | --- |
| common-REVERSE | CAGTGCAGGGTCCGAGGT |
| U6-RT-primer | GTCGTATCCAGTGCAGGGTCCGAGGTATTCGCACTGGATACGACAAAAAT |
| U6-FORWARD | GAAGATTTAGCATGGCCCCTGC |
| sja-bantam-RT-primer | GTCGTATCCAGTGCAGGGTCCGAGGTATTCGCACTGGATACGACACCAGC |
| sja-bantam-FORWARD | AGCAGGTGAGATCGCGATTAA |
| sja-let-7-RT-primer | GTCGTATCCAGTGCAGGGTCCGAGGTATTCGCACTGGATACGACACCACA |
| sja-let-7-FORWARD | ACAACAACGGAGGTAGTTCGT |
| sja-miR-2a-3p-RT-primer | GTCGTATCCAGTGCAGGGTCCGAGGTATTCGCACTGGATACGACCGTTCA |
| sja-miR-2a-3p-FORWARD | AAGCGCCTTCACAGCCAGTATT |
| sja-miR-2c-5p-RT-primer | GTCGTATCCAGTGCAGGGTCCGAGGTATTCGCACTGGATACGACCACATC |
| sja-miR-2c-5p-FORWARD | AACCATGACCCTTGTTCGACT |
| sja-miR-10-5p-RT-primer | GTCGTATCCAGTGCAGGGTCCGAGGTATTCGCACTGGATACGACCCAAAC |
| sja-miR-10-5p-FORWARD | AGCAACTTAACCCTGTAGACCCG |
| sja-miR-61-RT-primer | GTCGTATCCAGTGCAGGGTCCGAGGTATTCGCACTGGATACGACGAAGTG |
| sja-miR-61-FORWARD | AACACGTGTGACTAGAAAGTGCAC |
| sja-miR-124-5p-RT-primer | GTCGTATCCAGTGCAGGGTCCGAGGTATTCGCACTGGATACGACAAATCA |
| sja-miR-124-5p-FORWARD | AACTTGATCCATTTTCCGCGAT |
| sja-miR-125b-RT-primer | GTCGTATCCAGTGCAGGGTCCGAGGTATTCGCACTGGATACGACGAGCAA |
| sja-miR-125b-FORWARD | AAGCGACCTCCCTGAGACTGAT |
| sja-miR-190-5p-RT-primer | GTCGTATCCAGTGCAGGGTCCGAGGTATTCGCACTGGATACGACCACCAA |
| sja-miR-190-5p-FORWARD | GCGCGTGATATGTATGGGTTAC |
| sja-miR-3491-RT-primer | GTCGTATCCAGTGCAGGGTCCGAGGTATTCGCACTGGATACGACTGAACT |
| sja-miR-3491-FORWARD | ACGCCGTGAGCGATTACTG |
| sja-miR-3497-RT-primer | GTCGTATCCAGTGCAGGGTCCGAGGTATTCGCACTGGATACGACTGCCAT |
| sja-miR-3497-FORWARD | AGATCGATCGCAAGGGACTAC |
| sja-miR-3499-RT-primer | GTCGTATCCAGTGCAGGGTCCGAGGTATTCGCACTGGATACGACAAAATC |
| sja-miR-3499-FORWARD | ACCTCATATCTGAATCCGTGCT |
| sja-miR-3502-RT-primer | GTCGTATCCAGTGCAGGGTCCGAGGTATTCGCACTGGATACGACAAGACA |
| sja-miR-3502-FORWARD | AAGCGGAGTGACGATCGTAC |
| sja-miR-8185-RT-primer | GTCGTATCCAGTGCAGGGTCCGAGGTATTCGCACTGGATACGACAATGCT |
| sja-miR-8185-FORWARD | AACCGGAGGATCGATGAACG |
| SjPSMD-RT-F | CCTCACCAACAATTTCCACATCT |
| SjPSMD-RT-R | GATCACTTATAGCCTTGCGAACAT |
| Sjc_0000271-RT-F | CGCTTCGCTGTTCGGGATATG |
| Sjc_0000271-RT-R | TGTATTTCTTCTTCGCAACTTTCTGTG |
| Sjc_0003845-RT-F | ACTGAAGAGGAAATAGAACGCTTACG |
| Sjc_0003845-RT-R | CCACACCACCAACTAGCAATAGAAG |
| Sjc_0008865-RT-F | GACAACGGGACTTAAAAGAGAAACAAC |
| Sjc_0008865-RT-R | AGGCGAAGATGAAAGGGAACAAATC |
| Sjc_0001412-RT-F | GCCTTGACCGTTCAGTTCATACAG |
| Sjc_0001412-RT-R | AGCAGTGTCCAGAATTTCTAGCATAC |
| Sjc_0006748-RT-F | AGTTGGCTTTCATGTTACTGATCTCTC |
| Sjc_0006748-RT-R | TCTTCCGTCGCTGGTGTTGG |
| Sjc_0009104-RT-F | ACATCATTTAACCACTGAATCTACACG |
| Sjc_0009104-RT-R | TTTAGCACGCATACGCATTTGAATAG |
| Sjc_0008711-RT-F | CTCAGGCTATTCTTGAAGATCATTGC |
| Sjc_0008711-RT-R | ACCACTTCCATCACCGCTACC |
| Sjc_0004568-RT-F | AGGTCAAGAGCGTTTTCGTTCTATG |
| Sjc_0004568-RT-R | TGCGTCAGATAACCAATAGCCAAG |
| Sjc_0007166-RT-F | TCACGACTACAACAAAGGGTATAGC |
| Sjc_0007166-RT-R | GATGATCTTGCTTGGATTGATTGTGG |
| Sjc_0006400-RT-F | CAGCCAACGAGTCTAACACAAGG |
| Sjc_0006400-RT-R | GTAATAAGGTGGAGGAGCGTAAGC |
| Sjc_0007733-RT-F | GTTCGGACATCGTAGCACCTATC |
| Sjc_0007733-RT-R | TTGGAAGTATCAGGGAGATCAAGTTTG |
| Sjc_0000427-RT-F | GCATCCAACGAGTAACTGTAATCAATC |
| Sjc_0000427-RT-R | ATTCCTCTTCCTCCTTCACAACAAC |
| Sjc_0000962-RT-F | CCGCAAGGCTGATGAGATGTC |
| Sjc_0000962-RT-R | TACTGGCATTGTCGAAGGAATCTTG |
| Sjc_0006916-RT-F | AAGGACATTGAGCGAACAGAAGTAG |
| Sjc_0006916-RT-R | CTCCGATTCTTCAACCAGACTAGC |
