# Supplementary material for: Comparative microRNAs profile of Schistosoma japonicum male worms derived from single-sex and bisexual infections: Implications of the multifunctional role of microRNA
Source: Parasitol Res. 2025 Apr 24;124(4):43. doi: 10.1007/s00436-025-08489-x (PMC12021732; doi:10.1007/s00436-025-08489-x)
Supplement: Supplementary file 3 — Supplementary file3 (DOCX 20 KB) [file 436_2025_8489_MOESM3_ESM.docx]

**Comparative microRNAs profile of *Schistosoma japonicum* male worms derived from single-sex and bisexual infections: Implications of the multifunction role of microRNA**

Haoran Zhong^1^, Danlin Zhu^1^, Bowen Dong^1^, Luobin Wu^1,2^ Ke Lu^1^, Zhiqiang Fu^1^, Jinming Liu^1^, Guiquan Guan^3,4^, Yamei Jin^1,*^

^1^ National Reference Laboratory for Animal Schistosomiasis, Key Laboratory of Animal Parasitology of Ministry of Agriculture and Rural Affairs, Shanghai Veterinary Research Institute, Chinese Academy of Agricultural Sciences, Shanghai, P.R. China

^2^ College of Life Sciences, Shanghai Normal University, Shanghai, P.R. China

^3^ State Key Laboratory for Animal Disease Control and Prevention, College of Veterinary Medicine, Lanzhou University, Lanzhou Veterinary Research Institute, Chinese Academy of Agricultural Sciences, Lanzhou, Gansu, China

^4^ Key Laboratory of Veterinary Parasitology of Gansu Province, Gansu Province Research Center for Basic Disciplines of Pathogen Biology, Lanzhou, Gansu, China

^*^ Correspondence: [yameijin@shvri.ac.cn](mailto:yameijin@shvri.ac.cn)

**Table S2 DEMs of 28-day MM and SM**

| miRNA names | 28-day MM mean CPM | 28-day SM mean CPM | log2FC(SM/MM) | P-value | Adjust P-value |
| --- | --- | --- | --- | --- | --- |
| sja-miR-3502 | 8.4 | 0.1 | -3.09516 | 2.54E-22 | 9.00E-21 |
| sja-miR-8185 | 4.8 | 0 | -2.53605 | 1.32E-14 | 3.12E-13 |
| sja-miR-3491 | 2.2 | 0 | -1.67807 | 1.76E-07 | 2.08E-06 |
| sja-miR-3488 | 16.3 | 9.6 | -0.70671 | 3.26E-08 | 5.79E-07 |
| sja-miR-3482-5p | 0.6 | 0 | -0.67807 | 0.013388 | 0.059212 |
| sja-miR-3482-3p | 1.1 | 0.4 | -0.58496 | 0.011256 | 0.057082 |
| sja-miR-3483-3p | 0.8 | 0.2 | -0.58496 | 0.022741 | 0.080731 |
| sja-miR-2e-3p | 30.5 | 22.6 | -0.41656 | 1.17E-07 | 1.66E-06 |
| sja-miR-125b | 775538.2 | 619897.1 | -0.32317 | 9.53E-36 | 6.76E-34 |
| sja-miR-277 | 79.6 | 101.4 | 0.345364 | 0.004513 | 0.024647 |
| sja-miR-124-3p | 375.1 | 506.8 | 0.433144 | 0.013722 | 0.059212 |
| sja-bantam | 5930.3 | 8025.9 | 0.436495 | 0.030268 | 0.102335 |
| sja-miR-3479-3p | 1736.9 | 2419.3 | 0.477841 | 0.036834 | 0.118873 |
| sja-miR-71b-5p | 16400.2 | 33876.3 | 1.046518 | 0.002566 | 0.016561 |
| sja-miR-3492 | 57 | 119.5 | 1.054908 | 0.022185 | 0.080731 |
| sja-miR-10-5p | 2820.1 | 6531.4 | 1.211355 | 0.00015 | 0.001333 |
| sja-miR-3499 | 1.6 | 5.5 | 1.321928 | 0.044681 | 0.13793 |
| sja-miR-3497 | 0.5 | 3.2 | 1.485427 | 0.003142 | 0.018591 |
| sja-miR-124-5p | 9.9 | 30.7 | 1.540155 | 0.000501 | 0.003952 |
| sja-miR-2c-5p | 12.5 | 41.9 | 1.668018 | 1.36E-05 | 0.000137 |
